# Prevalence and risk factors associated with tungiasis in Mayuge district, Eastern Uganda

**DOI:** 10.11604/pamj.2016.24.77.8916

**Published:** 2016-05-24

**Authors:** Solomon Tsebeni Wafula, Charles Ssemugabo, Noel Namuhani, David Musoke, John Ssempebwa, Abdullah Ali Halage

**Affiliations:** 1 Department of Disease Control and Environmental Health, School of Public Health, College of Health Sciences, Makerere University, Kampala, Uganda

**Keywords:** Jigger, jigger infestation, prevalence, household, Uganda

## Abstract

**Introduction:**

Tungiasis is an endemic but neglected health problem in Uganda especially in resource poor communities. It is largely affecting rural communities in the Eastern, West Nile and Central regions. This study assessed prevalence and risk factors associated with tungiasis in Mayuge district, Eastern Uganda.

**Methods:**

This was a cross sectional study that used a semi-structured questionnaire and observational checklist to collect quantitative data from 422 households in 12 villages. Prevalence of tungiasis was defined as presence of Tunga penetrans in the skin of any household member at the time of data collection.

**Results:**

The prevalence of tungiasis was 22.5%. However, a big percentage 41.5% of households were reported to have had T. penetrans in the previous month while 49.5% had T. penetrans for more than one month. Majority (90.5%)of the participants used a pin, needle, or thorn to remove sand flea from infected body parts. Having dirty feet (AOR 3.86, CI (1.76-8.34)), dirty clothes (AOR 3.46, CI (2.00-5.97)), cracked house floor (AOR =6.28, CI (3.28-12.03)), dirty floor (AOR 3.21, CI (1.38-7.46)), littered compounds (AOR= 2.95, CI (1.66-5.26)) and rearing cattle (AOR 2.38, CI (1.28-4.45)) were associated with tungiasis. However, practicing preventive measures (AOR 0.51, CI (0.29-0.90)) was found protective for disease.

**Conclusion:**

Tungiasis is still a prevalent health problem in rural communities in Eastern Uganda due to a number of individual (host) and environmental factors. There is need to increase awareness regarding improvement in sanitation and hygiene to enable communities’ implements interventions for prevention of *T. penetrans*.

## Introduction

Tungiasis, also called Tunga penetrans infestation, is a parasitic skin infestation due to penetration of a female sand flea (*Tunga penetrans*) into the skin of its host [1]. It is one of the neglected tropical parasitic diseases [2] and has remained an important public health problem especially among economically challenged communities in sub-Saharan Africa, Latin America and the Caribbean [3]. Jigger infestation is endemic in developing countries, particularly where poverty and low standards of basic hygiene exist [4, 5]. *Tunga penetrans* in communities could be attributed to: presence of animal reservoirs such as dogs, cats, pigs, cattle, sheep, horses, mules, rats, mice and wild animals in close vicinity to living quarters, unpaved streets, illiteracy, ignorance and negligence [3], earthen floor houses, and walking bare footed or only with slippers [3, 6]. Poverty and prolonged dry spells [7] are presumably the other factors favouring the high prevalence of tungiasis among communities [3]. In most of the endemic areas, *T. penetrans* prevalence ranges from 15-40% [8] but at times can be as high as 50% in some rural communities [9]. Additionally, the prevalence of *T. penetrans* is higher in certain populations especially among certain age groups such as those between 20-60 years [10]. People afflicted with tungiasis are at increased risk of getting open wounds and suffer from anaemia and tetanus [1]. Other medical complications resulting from sand flea infestation such as inflammation, ulcerations, fibrosis, lymphangitis, gangrene and sepsis may emerge as secondary infections [11]. Social effects on affected persons include: low school attendance, discomfort, and poverty which leave people economically unproductive [12]. Surgical extraction of embedded sand fleas for example is a laborious process that wastes a lot of time which would be used in doing other economically productive activities. This further perpetuates poverty within the infested communities [13]. Uganda is one of the countries affected by tungiasis, especially in the rural communities of Eastern region [14, 15], West Nile districts and in some areas in Central region. However, the problem is more common in Eastern region where it is estimated that at least 2.4 million people are at risk of tungiasis [15]. Between June and October 2010, there was a rise in *T. Penetrans* infestation in the Eastern region. More than 300 families in Bukabooli, Mpungwe and Bukatube sub counties in Mayuge district ravaged by ailment and 3 people were reported dead [16]. The Ministry of Health in partnership with several non-governmental organizations (NGOs) launched a national anti-Jigger campaign worthy US $160,000 in a year [17]. The campaign involved interventions such as surgical removal of *T. penetrans* and treating infections with antiseptics and antibiotics. Others like health education campaigns on good sanitation and hygiene were also carried out. These interventions were implemented by Busoga Trust a local NGO [17]. In addition, in 2013 Mayuge district health authorities also intervened with US $72,000 to reward volunteers and health teams who wereadministeringhealth education and treatment to affected and infested communities [18]. However, despite all the above interventions, tungiasisis still prevalent in the area [19]. There is limited information on the prevalence of *Tunga penetrans* infestation and associated risk factors in Uganda. Therefore, this study sought to assess the prevalence and risk factors associated with tungiasis in order to inform about modifiable risk factors that should be addressed by interventions to prevent and control the disease.

## Methods

**Study design and setting:** This was a cross sectional household survey that utilized quantitative techniques of data collection. It was aimed at identifying the prevalence and risk factors associated with tungiasis among residents of Bukatube sub-county. Bukatube sub-county is located in Mayuge district in eastern Uganda. It was selected due to the repeated occurrence of *T. penetrans* in the area. It has 5 parishes and 32 villages with a population of 41,109 people, 8156 households and an average household size of 5.2 [20]. The main economic activity carried out in the sub county is subsistence farming. Otheractivities in the area include fishing, transport business and operating saw mills. The sub-county's main source of revenue is local tax revenue and donor funding. Firewood and charcoal are the major sources of energy in this community. The study participants were household heads who had lived in the area for at least one year (who responded to the questionnaire) and other household member who were observed for *T. penetrans* lesions. Household heads who were not available at the time of the interview with no any other adult present were not interviewed and their members were not observed.

**Sample size determination and sampling:** A sample size of 422 households was determined using a formulae for cross-sectional studies [21] at a 95% confidence interval and a 50% prevalence of *T. penetrans* since there were no studies that had been carried out on prevalence of tungiasis in a similar setting. A sampling error of 5% and a non - response rate of 10% were also used. A two stage cluster sampling technique was employed to identify parishes and villages where the study was carried out. At parish level, 3 out of the 5 parishes in the sub county were selected randomly. All names of parishes were written down on small pieces of paper, one paper was picked at a time without replacement until 3 parishes were obtained. At village level, 4 villages were randomly selected from each selected parish. All names of villages in a selected parish were written down on small pieces of paper. Four (4) villages were picked one at a time without replacement to make a total of 12 villages involved in the study. From each selected village, 35 households were systematically selected except one where 37 more households were selected in order to attain the sample size. A list of households obtained from respective local council one office was used as the sampling frame for the systematic sampling. The first household was picked randomly and the rest were selected usinga sampling interval based on the number of households in each village.

**Data Collection:** Data was collected using a semi-structured questionnaire and observational checklist. The questionnaire and checklist were developed with reference to literature on tungiasis [4, 10, 22–27]. The questionnaire was used to collect data on prevalence of *T. penetrans*, socio-demographic characteristics, knowledge on tungiasis and prevention, and individual (host) factors [28] associated with tungiasis. Prevalence of tungiasis was defined as presence of *T. penetrans*, in the skin of any household member at the time of data collection. It was ascertained by asking and confirmed by observing whether the respondent or other members in the household had any lesions on their limbs due to *T. penetrans* infestation. The checklist was used to collect information of environmental factors [28] in households associated with *T. penetrans*. Data collection tools were pretested in Bukatube trading center which was not involved in the study, and research assistants were trained on appropriate data collection techniques.

**Data analysis:** Data was entered and cleaned using Epi Info Version 7.0 (CDC; Atlanta)and analyzed using Stata 12.0 statistical software (Statacorp Texas; USA). Analysis was done at univariate, bivariate and multivariate levels. At univariate level, frequencies and proportions of study variables were computed for prevalence of *T. penetrans*. At bivariate level, individual and environmental factors were analysed to identify factors associated with tungiasis. Crude odds ratios (COR) and 95% confidence intervals were used as a test for association. All variables that were statistically significant at p ≤ 0.05 and those with biological plausibility were entered into a multivariable binary logistic regression model to identify independent predictors of tungiasis. Adjusted odds ratio (AOR) and 95% confidence intervals were presented after controlling for one another.

**Ethical considerations:** Approval to conduct this study was obtained from Makerere University, School of Public Health Higher Degrees Research and Ethics Committee and Uganda National Council for Science and Technology. Permission was also sought from Mayuge District Health Office and the local authorities of Bukatube sub-county before conducting the study. Written informed consent of all participants was obtained before data collection. Confidentiality was maintained for information collected from all study participants. Participants’ involvement in the study was voluntary. Each respondent was informed about the objective of the study, and privacy during administering of the study tools was ensured.

## Results

### Socio demographic characteristics of respondents

A total of 422 households participated in the study. More than half of the participants 55.7% (235/422) were females. The mean age of the participants was 38.9 years (standard deviation (SD) 15.0). Majority of the participants were married 80.6% (340/422), Christian 61.6% (260/422), working as farmers 76.3% (322/422) and earning a monthly income of 20 US$ and below 82.9% (350/422). Most of the participants either had never attended school 37.2% (157/422) or had attended primary education 46.9% (198/422) (Table 1).

**Table 1:** Socio demographic characteristics of respondents

Variables	Frequency (n = 422)	Percentage (%)
**Sex**		
Female	235	55.7
Male	187	44.3
Age mean (±SD)	38.9 (15.0)	
**Education level**		
did not attend school at all	157	37.2
Primary	198	46.9
Secondary	67	15.9
**Marital status**		
Single	35	8.3
Married	340	80.6
Widowed/separated/divorced	47	11.1
**Religion**		
Christian	260	61.6
Moslem	162	38.4
**Occupation**		
Unemployed	95	22.5
Farmer	322	76.3
Civil servant	5	1.2
**Monthly income**		
20 US$ and below	350	82.9
Above 20 US$	72	17.1

### Prevalence, knowledge and perceptions on prevalence of tungiasis

At the time of data collection, only 22.5% (95/422) of the households had *T. penetrans*. However, the number of households reported to have had tungiasis in the previous month were 41.5% (175/422). Nearly half 49.2% (87/177) of the households were reported to have had T. penetrans infestation for more than a month. More than half 55.7% (235/422) of the participants thought that *T. penetrans* were not a common problem in the community. A bigger percentage perceived infested individuals as either poor 32.2% (136/422) or lazy 30.8% (130/422) (Table 2). Majority 90.5% (382/422) of the participants used a pin, needle or thorn to remove T. penetrans from infected body parts. A big percentage 60.2% (254/422) of participants mentioned practicing preventive measures for *T. penetrans* such as good sanitation and hygiene, regular checking of the body for sand flea lesions and surgical removal, smearing houses with dug or clay to avoid dust and wearing shoes (Table 2).

**Table 2:** Prevalence, knowledge and perceptions on prevalence of tungiasis

Variable	Frequency (n)	Percentage (%)
**Household infested with Tunga penetrans (n = 422)**		
No	327	77.5
Yes	95	22.5
**Household infested with Tunga penetrans in past one month (n = 422)**		
No	247	58.5
Yes	175	41.5
**Infestation period (n = 177)**		
Less than a month	90	50.8
More than a month	87	49.2
**Tunga penetrans a common problem (n =422)**		
No	235	55.7
Yes	187	44.3
**Perceptions aboutinfested individuals or families (n = 422)**		
Are lazy	130	30.8
Are economically challenged	136	32.2
Cursed families	84	19.9
Irresponsible	72	17.1
**interventions and treatment of tungiasis (n = 422)**		
Chemicals like paraffin	23	5.5
Using a pin, needle, thorn	382	90.5
Household heads do not know any intervention	17	4.0
**Practicing preventivemeasuresfor tungiasis (n = 422)**		
No	168	39.8
Yes	254	60.2

### Individual factors associated with prevalence of tungiasis

Practicing preventive measures (aOR = 0.51:95% CI = 0.29-0.90) was 51% times more likely to protectpeople against tungiasis. Concerning personal hygiene, participants with dirty feet (aOR = 3.86: 95% CI = 1.76-8.34) were 3.9 times more likely to be infested with *T. penetrans* and those with dirty clothes (aOR = 3.46: 95% CI = 2.00-5.97) were 3.5 times more likely to be infested with *T. penetrans* (Table 3).

**Table 3:** Bivariate and multivariate analysis for individual factors associated with prevalence of tungiasis

Characteristic	Category	n (%)	cOR (CI)	aOR (CI)
**Sex**	Female	235 (55.7)	1	
	Male	187 (44.3)	0.99 (0.63-1.56)	
**Monthly income**	20 US$ and below	350 (82.9)	1	1
	Above 20 US$	72 (17.1)	0.38(0.17-0.82)**	0.72 (0.29-1.76)
**Education level**	Did not attend to school at all	157 (37.2)	1	1
	Primary	198 (46.9)	0.65 (0.40-1.06)	1.08 (0.61-1.91)
	Secondary and above	67 (15.9)	0.38 (0.18-0.84)*	1.34 (0.52-3.46)
**Marital status**	Single	35 (8.3)	1	
	Married	340 (80.6)	0.56 (0.21-1.49)	
	Widowed/separated/ divorced	47 (11.1)	1.15 (0.57-2.33)	
**Religion**	Christian	360 (61.6)	1	
	Moslem	162 (38.4)	0.92 (0.57-1.47)	
**Occupation**	Unemployed	95 (22.5)	1	
	Farmer	322 (76.3)	1.19 (0.13-10.84)	
	Civil servant	5 (1.2)	1.07 (0.11-10.08)	
**Knowledge on causes of tungiasis**	Economically challenged	44 (10.4)	1	
	Poor hygiene	270 (64.0)	0.28 (0.17-0.47)***	0.61 (0.33-1.13)
	Household heads don't know	108 (25.6)	0.76 (0.36-1.60)	1.34 (0.56-3.21)
**Practicing prevention measures**	No	168 (39.8)	1	1
	Yes	254 (60.2)	0.31 (0.19-0.49)***	0.51 (0.29-0.90)*
**Personal hygiene**	**Dirty feet**			
	No	196 (46.5)	1	
	Yes	226 (53.5)	8.90 (4.68-16.94)***	3.86 (1.76-8.34)***
	**Dirty clothes**			
	No	297 (70.4)	1	
	Yes	125 (29.6)	5.37 (3.30-8.74)***	3.46 (2.00-5.97)***
	**Long nails**			
	No	384 (91.0)	1	
	Yes	38 (9.0)	1.67 (0.81-3.46)	
	**Walking barefooted**			
	No	177 (41.9)	1	
	Yes	245 (58.1)	5.24 (2.90-9.47)***	1.71 (0.82-3.55)

COR = Crude odds ratio, AOR = Adjusted odds ratio, CI = Confidence Interval,

* p < 0.05

** p ≤ 0.01

*** p ≤ 0.001

### Environmental factors associated with prevalence of tungiasis

Households with cracked floor houses were 6times more likely to be infested with *T. penetrans* (aOR = 6.28:95% CI = 3.28-12.03) and those with dirty floors were 3 times more likely to be infested with *T. penetrans* (aOR = 3.21: 95% CI = 1.38-7.46). Individuals from households with littered compounds were also 3times more likely to suffer from tungiasis (aOR = 2.95: 95% CI = 1.66-5.26). Households whose members rear cattle were 2.4 times more likely to be infested with *T. penetrans* (aOR = 2.39:95% CI = 1.28-4.45) (Table 4).

**Table 4:** Bivariate and multivariate analysis for environmental factors associated with prevalence of tungiasis

Characteristic	Category	n (%)	cOR (95% CI)	aOR (95% CI)
Wall	**Cracked**			
	No	271 (64.2)	1	1
	Yes	151 (35.8)	3.94 (2.45-6.34)***	1.45 (0.74-2.83)
	**Rough**			
	No	115 (27.3)	1	1
	Yes	307 (72.7)	4.02 (2.01-8.06)***	0.74 (0.29-1.91)
	**Dirty**			
	No	207 (49.0)	1	1
	Yes	215 (51.0)	4.65 (2.73-7.91)***	1.63 (0.81-3.28)
Floor	**Dusty**			
	No	183 (43.4)	1	1
	Yes	239 (56.6)	4.73 (2.68-8.35)***	1.07 (0.50-2.29)
	**Cemented**			
	No	300 (71.1)	1	
	Yes	122 (28.9)	0.25 (0.13-0.50)***	
	**Earthen**			
	No	130 (30.8)	1	1
	Yes	292 (69.2)	4.93 (2.46-9.85)***	1.80 (0.77-4.21)
	**Cracked**			
	No	291 (69.0)	1	1
	Yes	131 (31.0)	9.83 (5.85-16.52)***	6.28 (3.28-12.03)***
	**Dirty**			
	No	158 (37.4)	1	
	Yes	264 (62.6)	6.24 (3.21-12.13)***	3.21 (1.38-7.46)**
Compound maintenance	**Littered**			
	No	243 (57.6)	1	1
	Yes	179 (42.4)	4.05 (2.48-6.60)***	2.95 (1.66-5.26)***
	**Dusty**			
	No	72 (17.1)	1	1
	Yes	350 (82.9)	2.29 (1.28-4.11)**	0.88 (0.37-2.09)
Animals present at home	**Pigs**			
	No	407 (96.5)	1	
	Yes	15 (3.5)	4.20 (1.48-11.91)**	3.58 (0.93-13.85)
	**Cattle**			
	No	191 (45.3)	1	
	Yes	231 (54.7)	1.76 (1.09-2.82)*	2.39 (1.28-4.45)**

COR = Crude odds ratio, AOR = Adjusted odds ratio, CI = Confidence Interval

* p < 0.05

** p ≤ 0.01

*** p ≤ 0.001

## Discussion

This study was carried out with the aim of determining the prevalence of tungiasis and associated risk factors in Bukatube sub-county, Mayuge district in Eastern Uganda. The study showed that the prevalence of tungiasis was high. This could be attributed to the poor hygiene, poverty and failure to seek treatment due to stigmatization. These results are consistent with data obtained from a study carried out among primary school pupils in Kenya [29]. However, finding from the current study were low compared to studies in Kenya and Tanzania [6, 22]. This may be due to the fact that the study was carried out during a low transmission season (rainy season) when the level of dust which provides an environment conducive *T. penetrans* survival had reduced. Our finding also corroborate with a study carried out in an endemic community in Brazil where infestations were low at the end of a rainy season and high at the peak of the dry season [30]. Our study also revealed thatmore families had suffered from *T. penetrans* in the previous month compared to those with the parasites at the time of the study. This shows that tungiasis is still a problem to the people of Bukatube sub-county unless interventions are increased. These study findings are consistent with findings from earlier studies conducted in *T. penetrans* endemic areas in Italy [31, 32]. However, the prevalence of tungiasis in this study is low compared to that reported in Nigeria and Cameroon [23, 26]. The high prevalence in the study conducted in Nigeria can partly be explained by the fact that it employed clinical examination to identify the infested individuals. This finding implies that the level of knowledge on prevention of *T. penetrans* and predisposing factors for tungiasis was relatively low. This could explain inability by the locals to take informed prevention and control measures for *T. penetrans*. However, these study findings contradict with a study conducted in Kenya where the reported level of knowledge on *T. penetrans* prevention was relatively high but there was no related evidence for sand flea prevention and control in the area [10]. This shows that there is need to increase awareness campaigns against tungiasis. Another important finding is that majority of the participants associated poor hygiene to tungiasis. This is consistent with previous literature that reported poor hygiene as the most important cause of *T. penetrans* infestation [10, 24]. It is therefore important that the hygiene status in homesteads is improved to reduce tungiasis [33]. Attainment of secondary level education was found to be protective for tungiasis. This finding is understandable as people with secondary and tertiary level of education are more informed and observe high levels of hygiene. These results match those that have been observed in earlier studies in Kenya [34, 35]. This finding emphasizes the importance of education and raising awareness in the prevention and control of *T. penetrans* [10] but also increasing income levels [34] since *T. penetrans* largely affect poor communities. However, several studies have also showed that tungiasis significantly leads to low school attendance, poor academic performance and high school dropout rates posing a big threat to children's education goals [25, 34, 36]. This study also discovered that practicing preventive measures was protective for *T. penetrans* infestation. This is because of preventive and curative measures employed by local authorities and NGOs helping families to make informed choices and change their behaviours. Personal hygiene practices such as having dirty feet, and putting on dirty clothes were found to be risk factors for tungiasis. Dirty feet and clothes provide a conducive environment for *T. penetrans* to survive and hide. Several studies have highlighted personal hygiene as an important factor in control and prevention of *T. penetrans* [7, 26, 34].

In our study, conditions related to poor housing were also associated with prevalence of tungiasis. Living in a house with cracked, rough and dirty walls, earthen, dusty, dirty and cracked floors and with littered and dusty compounds were found to be associated with tungiasis. Cracked house floors, dirty floors and littered compounds were the independent predictors tungiasis. Dusty surfaces, cracks and crevices in the walls and floors create a conducive environment for survival of sand flea. Therefore, walls should be plastered or smeared with cow dung or clay and floors should be cemented or smeared with cow dung or clay. The results in this study are similar to those from a study in Brazil, Nigeria, Kenya and Ethiopia where dusty and cracked floors were significantly associated tungiasis [29, 35, 37–40]. This highlights the fact that proper hygiene of houses is important in prevention of tungiasis. The free-living stages of *T. penetrans* usually develop in dry, cracked and sandy soil [23]. Houses with cracked earthen walls and floors promote multiplication of *T. penetrans*. Cracks and crevices in floors provide shelter for adult fleas until a suitable host presents [41]. However, houses with cemented floors are protective for *T. penetrans* infestation. They are always smooth and free from dust which hinders the development and survival of *T. penetrans*. This implies that cementing and smoothening floors of houses reduces the prevalence of *T. penetrans*. Littered compounds were significantly associated with prevalence of tungiasis. Other studies have also shown that compound maintenance is associated with *T. penetrans* infestation [10, 35, 42]. Littered compounds attract stray dogs, cats, and rodents which are important reservoirs for sand flea, and organic material contaminating the soil may provide a sheltered environment for the development of the free-living stages (larvae) of a sand flea. So, these study findings show the need for households to maintain general cleanliness of their compounds. Being a zoonotic disease, Tungiasis affects animals and humans alike. Among domestic animals dogs, cats, pigs, cattle, goats and others have been described to be commonly infested [43, 44]. Studies on the animal reservoirs of *T. penetrans* endemic communities in Brazil, Nigeria and Uganda showed that dogs, cats, pigs and goats were infested with *T. penetrans* [1, 23, 44]. In our study only cattle keeping was found to have an association with tungiasis. These results are in accord with a recent study which found living with cattle a risk factor for the disease [39]. These findings suggest that animals are resovoir hosts and risk factor for *T. penetrans*.

Study limitations: the study was carried out in a low transmission period (rainy season) which could have lowered the prevalence of tungiasis in the community. This being a cross sectional study, it was not easy to establish cause-effect relationship. In addition, lack of clinical examination to establish infested individuals may have increased response bias. Nevertheless, this study provides useful information on factors responsible for prevalence of tungiasis in a rural setting.

## Conclusion

*Tunga penetrans* are still a big problem in rural settings in Eastern Uganda. Interventions should therefore be put in place to prevent and control tungiasis. More emphasis should be given to improving personal hygiene and general cleanliness, housing structures and health educating the community on the risk factors of tungiasis and their prevention and control.

### What is known about this topic

- Tungiasisis a major health issue in Mayuge district, Eastern Uganda;
- Tungiasis is associated with low income levels among the victims.

### What this study adds

- Prevalence of tungiasis in Mayuge district, Eastern Uganda;
- Individual factors associated with tungiasis in Mayuge district, Eastern Uganda;
- Environmental factors associated with tungiasis in Mayuge district, Eastern Uganda.
